# Evaluation of a Topical Herbal Agent for the Promotion of Bone Healing

**DOI:** 10.1155/2015/905270

**Published:** 2015-02-25

**Authors:** Wing-Sum Siu, Chun-Hay Ko, Ka-Wing Lam, Elaine Wat, Wai-Ting Shum, Clara Bik-San Lau, Kam-Ming Ko, Leung-Kim Hung, David Tai-Wai Lau, Ping-Chung Leung

**Affiliations:** ^1^Institute of Chinese Medicine, 5/F, The CUHK Hong Kong Jockey Club School of Public Health Building, Prince of Wales Hospital, Shatin, New Territories, Hong Kong; ^2^State Key Laboratory of Phytochemistry and Plant Resources in West China, The Chinese University of Hong Kong, Shatin, New Territories, Hong Kong; ^3^Department of Orthopaedics and Traumatology, The Chinese University of Hong Kong, Shatin, New Territories, Hong Kong; ^4^Department of Chemical and Biomolecular Engineering, The Hong Kong University of Science & Technology, Clear Water Bay, Kowloon, Hong Kong; ^5^Division of Life Science, The Hong Kong University of Science & Technology, Clear Water Bay, Kowloon, Hong Kong

## Abstract

A topically used Chinese herbal paste, namely, CDNR, was designed to facilitate fracture healing which is usually not addressed in general hospital care. From our *in vitro* studies, CDNR significantly inhibited the release of nitric oxide from RAW264.7 cells by 51 to 77%. This indicated its anti-inflammatory effect. CDNR also promoted the growth of bone cells by stimulating the proliferation of UMR106 cells up to 18%. It also increased the biomechanical strength of the healing bone in a drill-hole defect rat model by 16.5% significantly. This result revealed its *in vivo* efficacy on facilitation of bone healing. Furthermore, the detection of the chemical markers of CDNR in the skin and muscle of the treatment area demonstrated its transdermal properties. However, CDNR did not affect the bone turnover markers in serum of the rats. With its anti-inflammatory and bone formation properties, CDNR is found effective in promoting bone healing.

## 1. Introduction

Fracture is the commonest problem in orthopaedic clinics. The estimated world incidence of adult fractures is around 9.0–22.8 per 1000 people per year [1]. Since fractures are more common among the elderlies, the fracture incidence is expected to further increase in the coming future due to the increasing longevity worldwide [2]. Patients with severe bone fractures are usually hospitalized. The median length of hospital stay after fracture fixation is 13.3 days for men and 19.6 days for women, and the maximum could be over 30 days for hip fractures [3, 4]. Obviously, fractures reduce the social productivities and intensify the social-economic burden [4–6].

Fracture management today is effectively done by surgeons and the healing outcome can be taken for granted. However, the healing process post-op is seldom addressed by hospital and clinical workers yet. The healing of the fracture relies mostly on self-recovery. Patients are usually left unattended except for the pain and inflammation control during hospitalization. In spite of many scientific researches which have been conducted to find the way to promote fracture healing including using biomaterial scaffolds [7–9], growth factors [10, 11], bone morphogenetic proteins [12–14], and biophysical stimulations [15, 16], these interventions have not been well accepted for routine clinical applications yet.

Herbal medicine has been used in China for thousands of years and modern researches have demonstrated its therapeutic effects on treating various diseases, including cancer [17]. More recently, its applications in modern Western therapy have also been discussed [18]. Facilitation of fracture healing is one of the major areas in traditional Chinese medicine. Traditionally, topical agents have been used to promote the healing of soft tissues and bone fractures. Nonetheless, the formulae of these herbal medicines are too diversified as yet. More importantly, the serious lack of relevant evidence-based scientific support and good systemic documentation of the clinical data makes them not well accepted worldwide.

The aim of this study is to investigate the efficacy of a topical herbal paste on the promotion of bone healing from an evidence-based scientific approach. Both* in vitro* and* in vivo* biological platforms will be used to verify the pharmacological properties essential for bone healing, namely, anti-inflammation, proangiogenesis, and cellular regeneration. The most novel component of the study lies in the confirmation of transcutaneous transport of the chemical compounds via the topical application.

Considering that over a hundred of medicinal herbs had been used historically in China for the treatment of skeletal injuries and bone fracture, we select the herbs for the present study according to the classical records and recent scientific papers testifying the pharmacological properties of the herbs related to fracture healing [19].

A herbal paste for topical use (CDNR) was thus created with four herbs, namely, Carthami Flos (C), Dipsaci Radix (D), Notoginseng Rhizoma (N), and Rhei Rhizoma (R). All of them were purchased from a reputable herbal supplier in Hong Kong. The identities of all herbs were authenticated using thin-layer chromatography with procedural references recommended by the Chinese pharmacopoeia [20]. The herbarium voucher specimens of the tested herbs were deposited in the museum bank of the Institute of Chinese Medicine, at the Chinese University of Hong Kong, with reference numbers as follows: Dipsaci Radix: 2010-3279; Rhei Rhizoma: 2010-3280; Notoginseng Rhizoma: 2010-3278; and Carthami Flos: 2010-3335.

## 2. Materials and Methods

### 2.1. Making of the Topical Agent

Extracts of CDNR were prepared via both aqueous and ethanol extractions. In the aqueous extraction, 250 g raw materials of each of the four herbs were cut into small pieces (C : D : N : R in 1 : 1 : 1 : 1 w/w) and were soaked in 1.0 L distilled water for 1 hour. They were boiled twice for 2 hours under reflux and then the aqueous extract was collected and filtered through a piece of absorbent gauze. The residue was further boiled with 95% ethanol for 2 hours under reflux. The ethanol extract was collected and filtered again. Both of the aqueous and ethanol filtrates were then concentrated at 50°C under reduced pressure separately, followed by lyophilizing into powder forms (CDNR(aq) and CDNR(e)) in a freeze dry system (Freezone 12, Labconco, Missouri, USA). The extraction yields of aqueous and ethanol extracts were 39.8% w/w and 5.0% w/w, respectively. The paste form of CDNR for topical use was completed by mixing 19.5 g CDNR(aq) and 3.0 g CDNR(e) with 17 mL 50% ethanol [19].

### 2.2. Chemical Composition of Topical Herbal Paste

The chemical composition of the herbal paste was determined by high-performance liquid chromatography-electrospray ionization-mass spectrometry (HPLC-ESI-MS) which included an Agilent 1290 Infinity LC System (Agilent Technologies, Santa Clara, California, USA), equipped with an online degasser, a binary pump, an autosampler, and Agilent 6410 Triple Quad LC/MS, and connected with Agilent MassHunter Workstation software. A Waters HSS T3 3.5 *μ*m (3.0 mm × 150 mm) HPLC column was used and kept at a temperature of 40°C. Eight chemical markers and samples were separated using a gradient mobile phase consisting of water (A) and acetonitrile (B). The gradient condition is 0 to 3 min, 10–27% B; 3 to 5 min, 27–33% B; 5 to 12 min, 33-33% B; 12 to 13 min, 33–80% B; 13 to 16 min, 80–90% B; 16 to 20 min, 90-90% B. The flow rate was set at 0.5 mL/min and the injection volume was 20 *μ*L.

MS analysis was performed on Agilent 6410 Triple-Quad LC/MS equipped with an ESI source [21] and monitored in negative ion mode and multiple reaction monitoring mode using target ions at* m/z* 611.2→325.0 for hydroxysafflor yellow A (HYA);* m/z* 285.0→117.0 for kaempferol (KAE);* m/z* 927.5→603.3 for asperosaponin VI (ASP);* m/z* 455.3→407.4 for oleanolic acid (OA);* m/z* 799.5→637.4 for ginsenoside Rg1 (Rg1);* m/z* 1107.6→119.0 for ginsenoside Rb1 (Rb1);* m/z* 269.0→241.0 for emodin (EMO), and* m/z* 283.0→239.0 for rhein (RHE). The abundance of the markers was determined quantitatively (Table 1).

All the chemicals were purchased from Sigma-Aldrich (St. Louis, Missouri, USA). Both methanol and acetonitrile were HPLC-grade (CHROMASOLV Plus, ≥99.9%). All the chemical markers were purchased from Tauto Biotech Co., Ltd (Shanghai, China). Their purities were over 97%.

### 2.3. *In Vitro* Studies

*In vitro* studies were targeting anti-inflammation and cellular proliferation.

#### 2.3.1. Cell Cultures

Murine monocyte/macrophage RAW264.7 and rat osteosarcoma UMR-106 cells were purchased from American Type Culture Collection (Manassas, VA, USA). RAW264.7 and UMR-106 cells were maintained in high-glucose DMEM (d-glucose: 3500 mg/L; GIBCO, USA), 10% v/v fetal bovine serum (FBS; Gibco, Gran Island, NY, USA), and 1% penicillin-streptomycin (PS; Gibco, Gran Island, NY, USA). All cells were maintained at 37°C, 5% CO_2_ humidified incubator.

*(i) Nitric Oxide (NO) Inhibitory Assay*. Macrophage cells RAW264.7 (4 × 10^5^ per well) were seeded. With 0.1 *μ*g of lipopolysaccharide (LPS) per mL of medium, CDNR(aq) or CDNR(e) was added (at concentrations ranging from 0 to 400 *μ*g/mL) and incubated for 24 h. The nitrite accumulation in the supernatant was determined by Griess reagent (Sigma, St. Louis, MO, USA) [22, 23] read at a wavelength of 540 nm. Cell viability of RAW264.7 in various concentrations of CDNR(aq) or CDNR(e) was determined by MTT assay (3-[4,5-dimethylthiazol-2-yl]-2,5-diphenyltetrazolium bromide) (Sigma, St. Louis, MO, USA) [22]. The relative amount of viable cells was determined using optical density at 540 nm and expressed as the percentage of Control samples without treatment.

*(ii) Determination of Production of Proinflammatory Cytokines Using ELISA Assay*. The RAW264.7 cell culture supernatants were subjected to test for the concentrations of cytokines, TNF-*α*, IL-6, and IL-1*β*, by enzyme-linked immunosorbent assay (ELISA) [22]. These assays were carried out according to the procedures recommended in the ELISA kit manual (BD Biosciences, San Jose, CA, USA).

*(iii) Osteoblast Proliferation and Viability Assays*. The osteoblast proliferation and viability were measured using 5-bromo-2′-deoxyuridine (BrdU) [24] incorporation and MTT cell viability assay [19, 25], respectively. Briefly, UMR-106 cells were seeded in DMEM with 10% v/v FBS. After 24 h, cells were exposed to different concentrations of CDNR(aq) or CDNR(e) in DMEM with 10% v/v FBS. An ELISA kit (Roche, Palo Alto, CA, USA) was used to determine the proliferation of the osteoblast in the BrdU incorporation assay. For the MTT assay, MTT solution (5 mg/mL) was added directly to the medium in each well, and the plate was then incubated at 37°C for 4 h. All medium was then aspirated and replaced with dimethyl sulfoxide (DMSO, Sigma-Aldrich, USA). The relative amount of viable cells was determined at optical density at 540 nm. The osteoblast proliferation and cell viability were expressed as the percentage of Control samples without treatment.

### 2.4. *In Vivo* Study

The animal model (rat) with artificially created bone defects via cortical and metaphyseal drillings is a recognized model for the study of bone healing [26, 27].

#### 2.4.1. Animals Models with Bone Injury

Twenty female Sprague-Dawley rats, aged 15.2 ± 1.41 months (mean ± standard deviation), were obtained from the Laboratory Animal Service Centre of the Chinese University of Hong Kong (CUHK). All of them were housed in a temperature-controlled (25°C) and light-controlled (12 h light/dark cycle) environment. All the animals were allowed access to standard rodent chow and water* ad libitum* throughout the study. After seven days of acclimatization, bone defects in the form of larger drill holes were created in the femur and tibia of the rats. The animal study had been approved by the Animal Experimental Ethics Committee of CUHK.

The rat was firstly anesthetized using a cocktail of ketamine and xylazine intramuscularly. On the left femur, two side-by-side drill holes (2 mm in diameter each) were created using an electric drill on the midshaft of the femur through an anterior-posterior approach. The two holes were connected to form a 2 mm × 4 mm defect using a dental milling burr. On the right tibia, (opposite leg) a bone defect, 2.4 mm in diameter, was made using the electric drill at the proximal metaphysis via a medial-lateral approach. All the drilled holes were cleaned thoroughly, irrigating with 0.9% sterile saline, making sure that small bone fragments would not remain.

The rats were divided into two groups with 10 rats each. In the control group (Control), the left femur and the right tibia were covered with just thin self-adhesive films without any treatment. In the herbal paste treatment group (CDNR), 0.5 mL CDNR paste was applied topically upon the left femur and right tibia, covering the operated sites. The paste was protected from falling off and drying with thin self-adhesive films. All the films and topical treatment paste were renewed at 2-day intervals. The whole treatment period lasted for 6 weeks.

#### 2.4.2. Assessments

*(i) Change in Microarchitecture Using Microcomputed Tomography (Micro-CT)*.* In vivo* longitudinal changes in bone volume (BV) of the drill-hole bone defect at the proximal tibia of the rat were monitored using micro-CT (MicroCT 40, Scanco Medical, Bassersdorf, Switzerland) within the experimental period. The rat was anesthetized using a cocktail of ketamine and xylazine intramuscularly and then fixed on a plastic holder to ensure a repeatable positioning. The right proximal tibia was scanned for 700 slices with energy 70 kVp and intensity 114 *μ*A. The isotropic resolution was 12.5 *μ*m per voxel. The volume of interest was determined within 250 continuous slices covering the whole drill hole. Segmentation parameters to define BV were fixed at sigma = 0.5, support = 1.0, and threshold = 245 for all analyses. These* in vivo* measurements were performed one day before the surgical drill-hole operation (Day −1), right after the operation (Day 0), and on 14, 28, and 42 days post-op (Day 14, Day 28, and Day 42, resp.).

*(ii) Bone Turnover Monitoring by Measuring Biochemical Markers*. Prior to microarchitecture measurement using micro-CT, blood was collected from the orbital venous plexus after the rat was anesthetized. Sera were obtained after centrifugation at 3000 rpm for 15 minutes at 4°C. They were then aliquoted and stored at −80°C before the biochemical markers were measured. Bone-specific alkaline phosphatase (BALP) was measured following the wheat germ lectin (WGL) (Sigma, St. Louis, MO, USA) precipitation method [28, 29]. Deoxypyridinoline (Dpd) was also measured using commercial available ELISA kit (MyBioSource, San Diego, USA).

*(iii) Biomechanical Bending Tests for Bone Strength Assessment*. The rats were euthanized on Day 42. Their left and right femora were harvested. Excessive soft tissue was removed but the periosteum was preserved. Four-point bending tests were performed using a Hounsfield material testing machine (KM25, Redhill, United Kingdom). A load cell with maximum 2500 N was mounted. The spans of the upper and lower supports were 8.0 and 20 mm, respectively. The drill-hole bone defect at the midshaft of the femur was located in the middle of the two upper supports and then the whole femur was loaded at a constant speed of 5 mm/min in posterior-anterior approach until failure. Load and work done at yield were recorded for analysis. All the data of the drilled femur (left) were normalized with the normal femur (right). The results were expressed as the normalized percentage based on the normal femur.

### 2.5. Testing for Transcutaneous Transport of the Topical Agent

In order to study whether the topical herbal materials go across the skin barrier, the presence and quantity of essential herbal chemical markers of the medicinal herbs used (CDNR) in the rat skin and underneath muscle were measured. 0.1 g of rat skin and muscle was collected from the region of the topical paste application after the rat was sacrificed. The tissues were rinsed with PBS gently and then homogenized with 1 mL of homogenization buffer (50 mM Tris, 1 mM DTT, and pH 7.5) using a homogenizer on ice. Then, 1 mL of acetonitrile was added to the homogenate and filtered for analysis. The presence of the chemical markers of the herbal compounds was revealed using the LC-MS equipment. The abundance of each marker was quantitatively determined.

### 2.6. Statistical Analysis

For the multiple group comparisons of cell proliferation, viability, NO, and cytokine production, the significance of the differences among the treatment groups and their respective control groups were tested by one-way ANOVA with Dunnett's post hoc test. For the* in vivo* studies, the differences between Control and the treatment group (CDNR) were analyzed with nonparametric Mann-Whitney *U* test. The results of bone microarchitecture (from micro-CT) and biochemical markers (BALP and Dpd) were analyzed following repeated measure ANOVA. Data were presented as mean ± standard deviation (SD) unless otherwise specified. All the statistical analyses were carried out using GraphPad Prism version 5.0 for Windows (GraphPad Software, San Diego, CA, USA) and tests were two-sided, with *P* < 0.05 being considered as statistical significant.

## 3. Results

### 3.1. *In Vitro* Anti-Inflammatory Effects of CDNR

**Figure 1:**
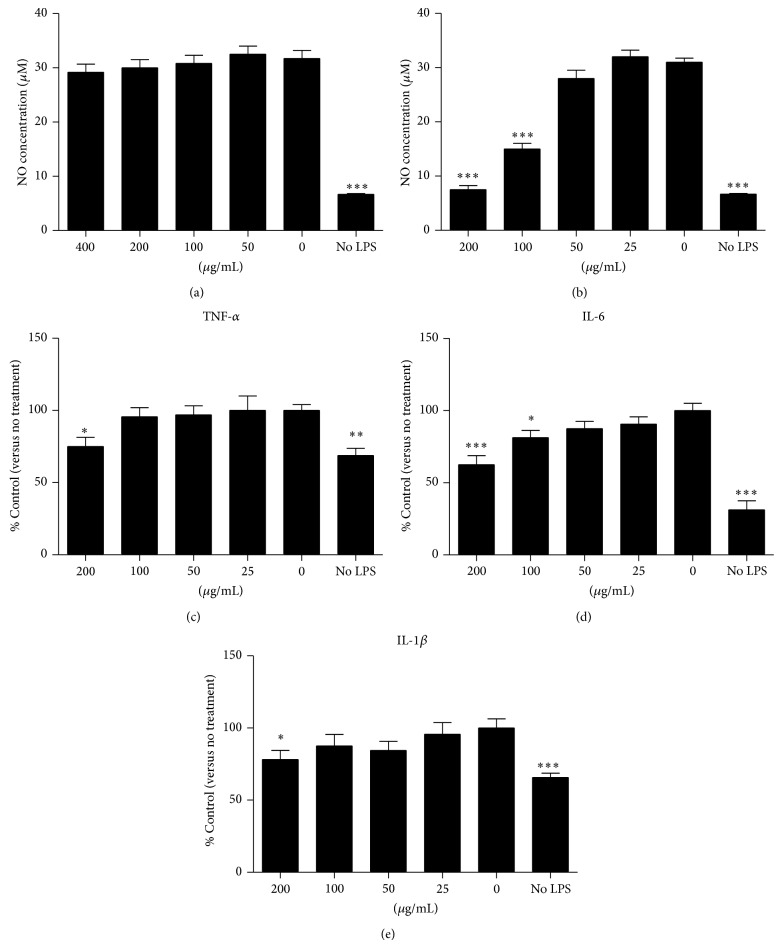
Anti-inflammatory effects of CDNR. (a, b) Effect of aqueous (CDNR(aq)) and ethanol (CDNR(e)) component of CDNR on nitric oxide (NO) production by RAW264.7 induced by LPS; (c, d, and e) Effect of CDNR(e) on TNF-*α*, IL-6, and IL-1*β* production by RAW264.7. Data are expressed as mean and standard deviation (error bar). ^*^ *P* < 0.05, ^**^ *P* < 0.01, and ^***^ *P* < 0.001 versus Control (0 *μ*g/mL).

Results demonstrated that CDNR(aq) did not affect the NO production in LPS-induced RAW264.7 cells (Figure 1(a)). In contrast, CDNR(e), at 100 *μ*g/mL and 200 *μ*g/mL, significantly suppressed the NO production in RAW264.7 cells after LPS induction by 51% and 77%, respectively (*P* < 0.001), when compared to the control group without herbal treatment (0 *μ*g/mL) (Figure 1(b)). However, CDNR(e) was nontoxic to the cells because it did not affect the normal cellular activity at concentration lower than 200 *μ*g/mL (data not shown). CDNR(e) also suppressed the proinflammatory cytokines production in RAW264.7 cells after LPS induction. At 200 *μ*g/mL, it effectively downregulated the production of TNF-*α* (by 25.2%, *P* < 0.05; Figure 1(c)), IL-6 (by 35.2%, *P* < 0.001; Figure 1(d)), and IL-1*β* (by 20.9%, *P* < 0.05; Figure 1(e)) when compared with its corresponding controls, which further indicated that CDNR(e) was anti-inflammatory.

### 3.2. *In Vitro* Osteogenic Effect of CDNR

**Figure 2:**
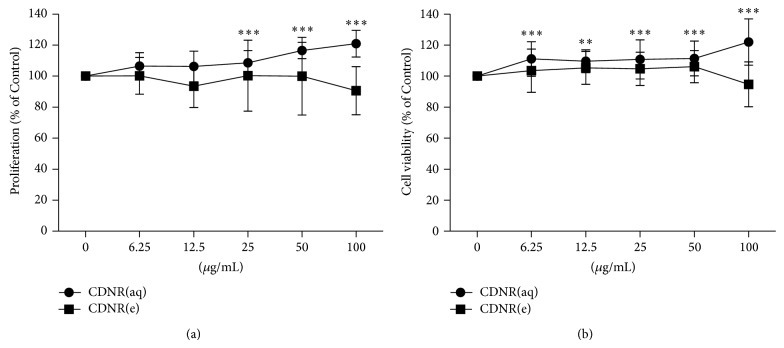
Osteogenic effect of CDNR on bone cells. (a) UMR106 proliferation at different concentration of CDNR assessed by BrdU assay; (b) UMR106 viability at different concentration of CDNR assessed by MTT assay. (CDNR(aq), CDNR(e)) Aqueous and ethanol component of CDNR. Data are expressed as mean and standard deviation (error bar). ^**^ *P* < 0.01, ^***^ *P* < 0.001 versus Control (0 *μ*g/mL).

As shown in Figure 2(a), after treatment of CDNR(aq) for 24 h, significant proliferative effects were observed in UMR-106 cells from 25 to 100 *μ*g/mL (*P* < 0.001) with increments of 10.8% to 22%. Similar significant cell viability-enhancement effects were observed in UMR-106 from 6.25 to 100 *μ*g/mL (Figure 2(b)). CDNR(e) did not affect UMR-106 cells after 24 h of treatment and up to 100 *μ*g/mL in both assays.

### 3.3. *In Vivo* Effect of CDNR on the Microarchitecture of Drilled Bone

From the quantitative micro-CT analysis at the drill-hole bone defects over the proximal tibial metaphyses of the rats, bone volume (BV) (from Day 0 to Day 42) was changed significantly (*P* < 0.001) depending on the time (number of days post-op) in both Control and CDNR groups. It changed from 15.43 mm^3^ to 17.11 mm^3^ in CDNR while from 15.05 mm^3^ to 18.02 mm^3^ in Control (Table 2). However, there was no significant difference in the BV between the CDNR and the control group when it was compared at each specific time point throughout the study. Since the femur was designed for biomechanical tests, no micro-CT data was obtained from the femur.

### 3.4. *In Vivo* Effects of Topical CDNR Application on Systemic Bone Remodeling

There was a significant overall increase in the bone-specific alkaline phosphatase (BALP) level in serum in both control and CDNR groups of the rats after the drill-hole bone defect had been created (*P* < 0.001). However, there was no significant difference between the two groups at each time point throughout the study (Table 3). On the other hand, an overall significant descending trend of serum deoxypyridinoline (Dpd) was observed after the surgery in both groups (*P* = 0.003). Similar to BALP, no difference of Dpd between the two groups at each time point was found neither (Table 3).

### 3.5. *In Vivo* Effect of CDNR on the Bone Strength (Biomechanical Properties) after Healing

**Figure 3:**
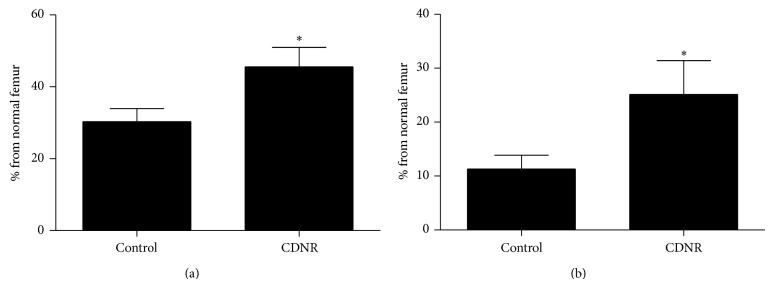
Biomechanical properties of femur with drill-hole bone defect. (a) Yield strength; (b) work done at yield strength. Data are expressed as mean and standard error of mean (error bar). ^*^ *P* < 0.05 versus Control.

Femora with cortical drill holes showed 16.5% higher normalized yield strength (% from the contralateral normal femur) in the CDNR treated group when compared with the Control (*P* < 0.05) after 42 days of treatment (Figure 3(a)). Similar findings were observed when the total work done at yield point was analyzed. CDNR treated bone showed 13.8% higher energy than the Control to reach the yield point (*P* < 0.05) (Figure 3(b)). The 4-point bending tests illustrated that the biomechanical properties of the healing at the drill-hole bone defects were improved significantly when the topical herbal paste was applied.

### 3.6. Transdermal Transport of the Herbal Compounds through the Rat Skin and Muscle

**Figure 4:**
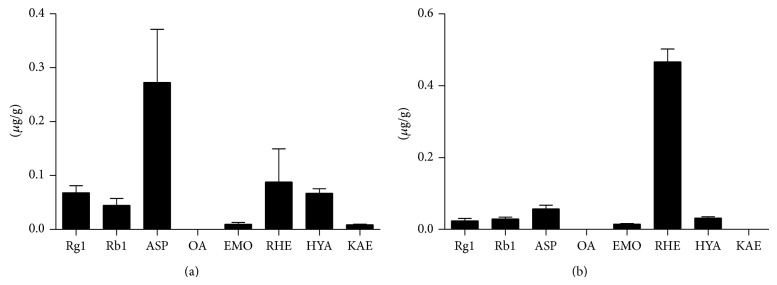
Concentration of the chemical markers of CDNR remained in the soft tissues of the rat. (a) Skin; (b) muscle. Data are expressed as mean and standard deviation (error bar). Rg1: ginsenoside Rg1; Rb1: ginsenoside Rb1; ASP: asperosaponin VI; OA: oleanolic acid; EMO: emodin; RHE: rhein; HYA: hydroxysafflor yellow A; KAE: kaempferol.

The transdermal transport of CDNR was determined by identifying the relevant chemical marker compounds in the skin and muscle after 6 weeks of herbal treatment using Q-TOF LC/MS technique. As shown in Figure 4(a), all the chemical markers of CDNR, except oleanolic acid (OA), were detected in the rat skin with asperosaponin VI (ASP) showing the most abundance. Similarly, those 7 chemical markers detected in the skin, except kaempferol (KAE), could also be found in the muscle. OA was nondetectable neither in the muscle. With a little bit difference from skin, rhein (RHE) but not the ASP was found to be the most abundant in the muscle (Figure 4(b)).

## 4. Discussion

Scientific studies on topical TCM treatment for fracture healing are seldom reported in international journals. As a result, there is lack of scientific evidence to support the use of topical herbal paste to facilitate fracture healing clinically. This study is one of the few scientific reports exploring the pharmacological activities of a topical agent from two standard directions, namely, direct cellular influences using standard cell lines for the study of anti-inflammation and cellular proliferation (*in vitro*) and life-animal (*in vivo*) situation of bone regeneration.

In the* in vitro* studies, the aqueous component of CDNR promoted the UMR-106 proliferation significantly. This observation indicated that CDNR could promote osteogenesis which must be an important mechanism during bone repair, particularly during the reparative phase of fracture healing when endochondral ossification takes places and osteoblasts start to form new lamellar bone on the cartilaginous callus [30].

Anti-inflammation is one of the key principles in the treatment of fractures as well as traditional bone setters' advocations. It controls the swelling and relieves the pain from the fracture site and the surrounding soft tissue which promotes overall healing. During inflammation, there is an increased production of various mediators such as arachidonic acid metabolites and cytokines. Besides, there are confirming evidences stating that nitric oxide (NO) produced by inducible NO synthase (iNOS) plays one of the important roles in inflammatory disorders such as rheumatoid arthritis [31, 32]. Therefore, chemicals that inhibit the NO production by iNOS in macrophages would be the potential treatments to reduce inflammatory responses [22]. In this regard, it is meaningful to examine the effects of CDNR on NO production. One of our* in vitro* studies demonstrated that the ethanol component of CDNR suppressed LPS-induced nitric oxide and cytokines production significantly. These observations indicated that there are active ingredients in CDNR that can suppress inflammation and thus controls the swelling and relieves the pain during fracture healing.

The ultimate goal of bone repair is to restore the bone strength of the injured bone to its original level without fracture or defect [33]. Assessment on the bone strength during fracture or defect healing in preclinical studies is essential to evaluate the effectiveness of an intervention on bone repair. The biomechanical tests of the current study showed strong evidences of healing promotion from the topical use of the herbal paste. The higher bending yield strength and work load ability of the femur in the CDNR group than in the control group revealed that the treatment elevated the strength of the bone that can tolerate much more stress and energy before permanent deformation which could be caused by microfractures. Perhaps the topical use of CDNR may reduce the risk of refracture during fracture healing due to insufficient structural remodeling [34]. CDNR, therefore, might act like some antiresorptive pharmaceutical agents when they help building up bone density and bone strength in the prevention of refracture [35].

Nowadays, microstructure of the trabecular bone is one of the most important parameters in orthopaedic researches. The microarchitecture of trabecular bone was highly correlated with bone strength [36, 37]. Therefore, assessment on microarchitecture gives a new dimension to researchers in terms of clinical observations of bone. In the micro-CT analysis of the current study, no difference in BV in the trabecular region of the tibia was found between CDNR and Control. It might be due to the fact that the bone healing process in trabecular metaphysis is different from that in cortical diaphysis. Healing of bone lesions involves many factors, including different processes according to the type of bone (cortical or cancellous), position and size of the wound, and the biomechanical environment provided onto the injured bone [38]. Diaphyseal defect on femur of mice was rapidly repaired while the epimetaphyseal defect failed to bridge the cortical gap even 13 weeks after surgery [39]. Another study reported that when the defect with the same size was located at the metaphysis of distal femur of mice, it bridged completely within 6 weeks [40].

Bone formation and bone resorption are two tightly coupled activities in a continuous and dynamic process of bone remodeling. During bone resorption, calcium and other matrix constituents are released into the bloodstream. When in bone formation, skeletal-specific proteins (enzymes or matrix components) can leach into the circulation. These biochemical markers can be measured by a noninvasive, systemic, and sensitive method to detect changes of bone turnover in a short interval of time [41]. These measurements have also been used to predict bone fracture [42, 43]. The bone formation marker BALP increased in both groups of rats in this study. This observation illustrated that the osteoblastic activity of the rats, whether Control or CDNR, was increased after the drill-hole bone defect had been created. The data was coherent with the results of a previous study on human, in which the increase of BALP started to be observed from the 7th day after fracture, elevated by nearly 200% to week 12, and was maintained at high level thereafter [44]. In the fracture of the tibial shaft, BALP increased at 4 weeks [45]. On the other hand, the serum concentration of the bone resorption marker Dpd was found reduced in both groups of rats post-op. It indicated that bone resorption activity was retarded during bone repair. Similar findings were also reported in the studies using bovine [46] and canine [47] model of bone fracture healing. They showed that the serum TRAP activity, another bone resorption biochemical marker, was low during the postfracture period. On the whole, there was a net bone gain after the systemic bone remodeling process of the rats in the processes of drill-hole bone repair. Apparently, the topical CDNR treatment did not alter this process systematically. Its promotional effect on bone healing could possibly be a local response.

The essential factor required for the efficacy of any topical treatment is its transdermal transport. The efficiency of the transport of the active ingredients of the drug depends on the physicochemical properties of the molecules, including the size (molecular weight), charge, and hydrophilicity (water solubility) [48–50]. In the transdermal experiment of this study, almost all the chemical markers of CDNR were detected by LC/MS in both the skin and muscle at the treatment sites of the rats. This observation illustrated that each herbal component of CDNR did pass through the skin barrier to cast its therapeutic effect. It further supports the hypothesis that the herbal components of CDNR influence bone healing via local therapeutic responses.

Topical agents have been popular before the present era of fracture management. Now that fracture treatment is well developed, clinicians have isolated themselves from the traditional treatment applications. As a matter of fact, since no scientific records are available neither with the biological activities nor with the more basic transdermal transport, the old practice might not deserve much attention.

However, since topical herbal agents are still widely used in the households and there might be special indications of utilization, it would be necessary to start serious scientific explorations. Indeed, some difficult fracture healings might still benefit from simple, harmless topical applications as long as toxicity and sensitivity could be ruled out. Moreover, the anti-inflammatory effects could also help with the recovering of soft tissue injuries [51].

## 5. Conclusions

The present study demonstrated for the first time that a 4-herb formula (CDNR) was effective in the control of inflammation and facilitation of bone regeneration when used topically. Its efficacy on the promotion of cortical bone repair was also revealed in the rat experiments. This study gave us sufficient scientific evidences that our novel herbal paste is a safe and promising supplement agent to facilitate bone healing under special circumstances.

## Figures and Tables

**Table 1:** Chemical composition of CDNR paste for animal studies.

Herbal source	Name of chemical marker	Content (mg/g of paste mixture powder)
Carthami Flos (C)	Hydroxysafflor yellow A (HYA) Kaempferol (KAE)	2.14 0.004

Dipsaci Radix (D)	Asperosaponin VI (ASP) Oleanolic acid (OA)	19.23 0.05

Notoginseng Rhizoma (N)	Ginsenoside Rg1 (Rg1) Ginsenoside Rb1 (Rb1)	2.00 5.5

Rhei Rhizoma (R)	Emodin (EMO) Rhein (RHE)	0.96 1.00

**Table 2:** Change of bone volume of the drill-hole bone defect.

	Control (mm^3^)	CDNR (mm^3^)	*P* value
Day −1	20.223 ± 1.589	19.509 ± 2.111	0.457
Day 0	15.045 ± 1.696	15.427 ± 2.247	0.707
Day 14	18.249 ± 1.766	18.091 ± 2.413	0.883
Day 28	17.582 ± 1.801	17.231 ± 2.180	0.730
Day 42	18.020 ± 1.949	17.111 ± 1.826	0.352

There was no significant difference in the bone volume (mm^3^) of the drill-hole bone defect in tibial metaphysis measured by using micro-CT between Control and CDNR at each time point. Day −1 and Day 0: measurement prior to and right after the drill-hole bone defect creation, respectively. Data are expressed as mean ± standard deviation.

**Table 3:** Change of biochemical markers in serum after drill-hole bone defects had been created.

	BALP (U/L)			Dpd (nmol/L)		
	Control	CDNR	*P* value	Control	CDNR	*P* value
Day −1	3.582 ± 1.207	3.473 ± 1.391	0.882	3.665 ± 1.421	2.862 ± 0.577	0.229
Day 14	4.887 ± 1.610	4.606 ± 1.649	0.762	2.964 ± 1.118	2.614 ± 0.710	0.532
Day 28	5.909 ± 2.235	5.120 ± 2.458	0.557	2.761 ± 1.657	2.303 ± 0.624	0.540
Day 42	4.991 ± 1.605	5.283 ± 2.153	0.785	2.791 ± 1.586	1.891 ± 0.411	0.208

There was no significant difference in the serum biochemical markers between Control and CDNR at each time point. Day −1: blood was collected prior to the drill-hole bone defect creation; BALP: bone-specific alkaline phosphatase; Dpd: deoxypyridinoline. Data are expressed as mean ± standard deviation.
